# Top-emitting 940-nm thin-film VCSELs transferred onto aluminum heatsinks

**DOI:** 10.1038/s41598-021-04625-6

**Published:** 2022-01-12

**Authors:** Sunghyun Moon, Yeojun Yun, Minhyung Lee, Donghwan Kim, Wonjin Choi, Ji-Yong Park, Jaejin Lee

**Affiliations:** 1 Department of Electrical and Computer Engineering, Ajou University, Suwon, 16499 South Korea; 2 Department of Physics and Department of Energy Systems Research, Ajou University, Suwon, 16499 South Korea; 3 RayIR Corporation, LTD, 156 Gwanggyo-ro, Yeongtong-gu, Suwon, 16506 South Korea

**Keywords:** Engineering, Optics and photonics

## Abstract

Thin-film vertical cavity surface emitting lasers (VCSELs) mounted onto heatsinks open up the way toward low-power consumption and high-power operation, enabling them to be widely used for energy saving high-speed optical data communication and three-dimensional sensor applications. There are two conventional VCSEL polarity structures: p-on-n and n-on-p polarity. The former is more preferably used owing to the reduced series resistance of n-type bottom distributed Bragg reflection (DBR) as well as the lower defect densities of n-type GaAs substrates. In this study, the p-on-n structures of thin-film VCSELs, including an etch stop layer and a highly n-doped GaAs ohmic layer, were epitaxially grown in upright order by using low-pressure metalorganic chemical vapor deposition (LP-MOCVD). The p-on-n structures of thin-film VCSELs were transferred onto an aluminum heatsink via a double-transfer technique, allowing the top-emitting thin-film VCSELs to keep the p-on-n polarity with the removal of the GaAs substrate. The threshold current (*I*_*th*_) and voltage (*V*_*th*_) of the fabricated top-emitting thin-film VCSELs were 1 mA and 2.8 V, respectively. The optical power was 7.7 mW at a rollover point of 16.1 mA.

## Introduction

With the demand on high-speed data communication networks, vertical-cavity surface-emitting lasers (VCSELs) are considered as a prominent light source that is strongly favored for use in optical data links due to their low-power consumption and high-modulation speed at low threshold current, along with a low circular beam divergence that enables efficient optical coupling with other systems^1–4^. The widespread adoption of VCSELs for a range of additional applications can be achieved with the development of high-power VCSELs. High-power VCSELs, especially those operating at a wavelength of 940 nm with a prominent dip in the solar spectrum, have recently attracted much attention in that they can be widely used for three-dimensional optical sensing systems and modern lidar with the time-of-flight (ToF) technique^5–7^.

However, there is a limitation of securing high-power VCSELs due to thermal resistance caused by the significantly increased temperature around the active layers at higher injection current^8,9^. The thermal resistance has adverse effects on device properties such as the threshold current, threshold voltage, output power, and emission spectrum^10^. Furthermore, the self-heating effect reduces the operating lifetime of VCSELs, which in turn makes the reliability of VCSELs unstable^11,12^. In order to handle this issue, much effort has been devoted to numerous attempts at transferring thin-film VCSELs onto heatsinks with the removal of the growth substrate, leading to the improved thermal characteristics of VCSELs^13–16^. The driving voltage of the substrate-removed thin-film VCSELs can be lowered due to the reduction of series resistance at the expense of considerably thick and moderately doped GaAs substrates^17^.

The high-power thin-film VCSELs require the structural design of distributed Bragg reflectors (DBRs). The VCSELs with p-on-n polarity, placing p-type DBRs above multiple-quantum wells (MQWs), are preferably used over those with n-on-p polarity owing to the reduced series resistance of n-type highly reflective DBRs as well as the lower defect densities of n-type substrates^18^. The n-type DBRs with high electron mobility and lateral conductivity promise to decrease the current crowding effects, which leads to lowered threshold voltage and device heating^19^. In order to allow the thin-film VCSELs transferred onto a heatsink to keep the conventional p-on-n polarity, the top-emitting thin-film VCSELs are required to be epitaxially grown in reverse order. However, it is worth noting that the inverted VCSEL structures are subjected to a thermal budget due to the thick n-type DBRs after the relatively thin p-type DBRs^20,21^. The thermal budget can raise the critical growth issues of VCSELs since tight control over thickness, composition and doping concentration for a large number of DBR periods is an indispensable prerequisite for achieving the necessary high reflectivity.

**Figure 1:**
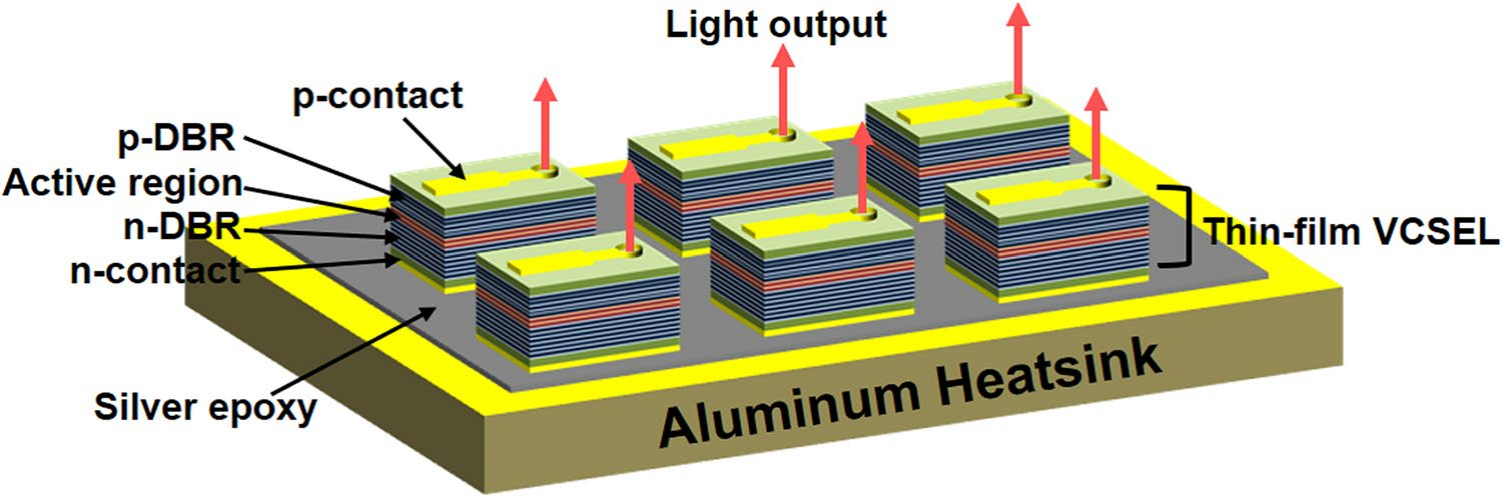
Schematic p-on-n structures of top-emitting 940-nm thin-film VCSELs transferred onto an aluminum heatsink. The top-emitting 940-nm thin-film VCSELs with p-on-n polarity are successfully fabricated onto an aluminum heatsink by using a double-transfer technique. The thin-film VCSELs with p-on-n polarity, placing p-DBRs above the active region, enable high-performance thin-film VCSELs owing to the reduced series resistance of n-DBRs as well as the lower defect densities of n-type substrates.

In the present paper, the p-on-n structures of top-emitting thin-film VCSELs, including an etch stop layer and a highly n-doped GaAs ohmic layer, are epitaxially grown in upright order using metalorganic chemical vapor deposition (MOCVD). A transfer approach to release and transfer the thin-film VCSELs from the growth substrate onto an aluminum heatsink is described in detail. The thermal conductivity of Aluminum (2.47 W/cm K), 4.57 times higher compared to that of a GaAs substrate (0.54 W/cm K), enables the high-power VCSELs operation due to the higher rollover current with the lowered thermal resistance. The top-emitting 940-nm thin-film VCSELs are successfully fabricated onto aluminum heatsinks via a transfer approach that utilizes a double-transfer process with a polyimide carrier, an aluminum heatsink, and adhesive materials such as wax, silver-filled epoxy. This double-transfer technique allows the top-emitting thin-film VCSELs to keep p-on-n polarity after transferring the upright-order VCSELs onto the aluminum heatsink. Figure 1 shows schematic top-emitting 940-nm thin-film VCSELs transferred onto an aluminum heatsink. The fabricated thin-film VCSELs are investigated using optical spectrum and light–current–voltage (L–I–V) curves.

## Results

### Double-transfer technique for top-emitting thin-film VCSELs mounted onto an aluminum substrate

**Figure 2:**
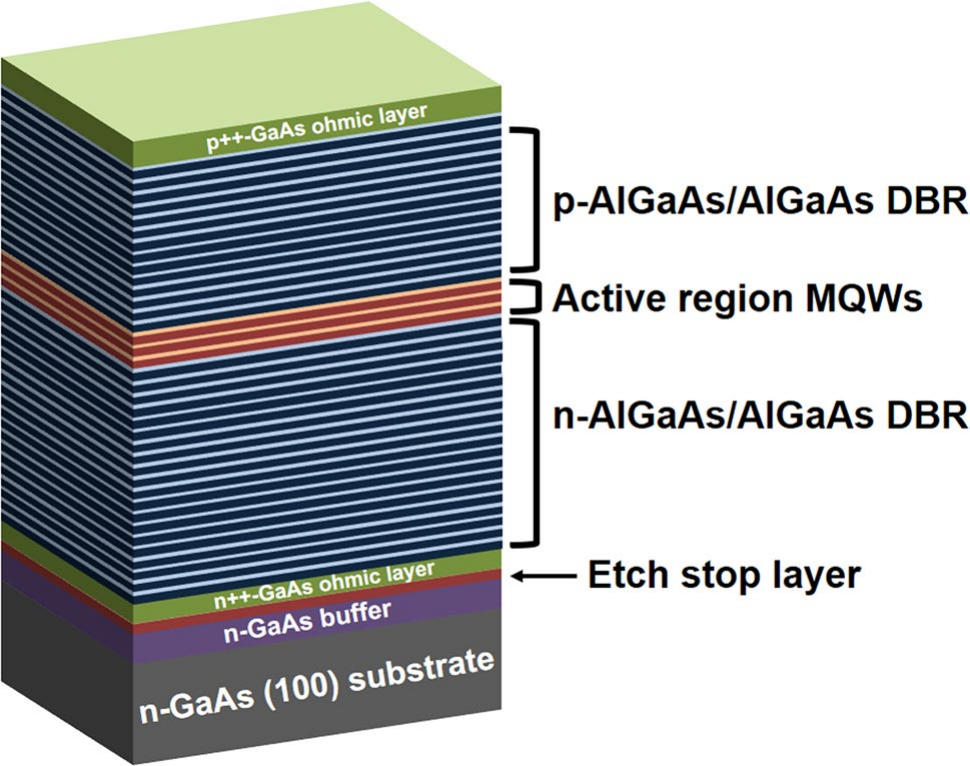
P-on-n structures of top-emitting 940-nm thin-film VCSELs. There is the etch stop layer inserted between the n-type GaAs ohmic layer and n-GaAs buffer in order to keep the epilayers of VCSELs intact during the removal of the n-GaAs substrate in the NH_4_OH-based etchant. The highly n-doped GaAs ohmic layer can improve the electrical characteristics of thin-film VCSELs with the removal of GaAs substrates.

The p-on-n structures of top-emitting 940-nm thin-film VCSELs are epitaxially grown on n-GaAs substrates in upright order using MOCVD, as shown in Fig. 2. Compared to the conventional p-on-n structures of bulk VCSELs, the p-on-n structures of thin-film VCSELs included additional layers, such as the etch stop layer and n-GaAs contact layer, while the p-GaAs contact layer, MQWs, two oppositely doped n-, and p-DBRs remained unchanged (see “Methods”). The etch stop layer was inserted to keep the epilayers of thin-film VCSELs intact during the removal of a GaAs substrate in NH_4_OH-based etchant. The heavily n-doped GaAs ohmic layer can enhance the electrical characteristics of thin-film VCSELs as a way of alleviating the electrical loss stemming from the considerably thick and moderately doped GaAs substrates^17^.

**Figure 3:**
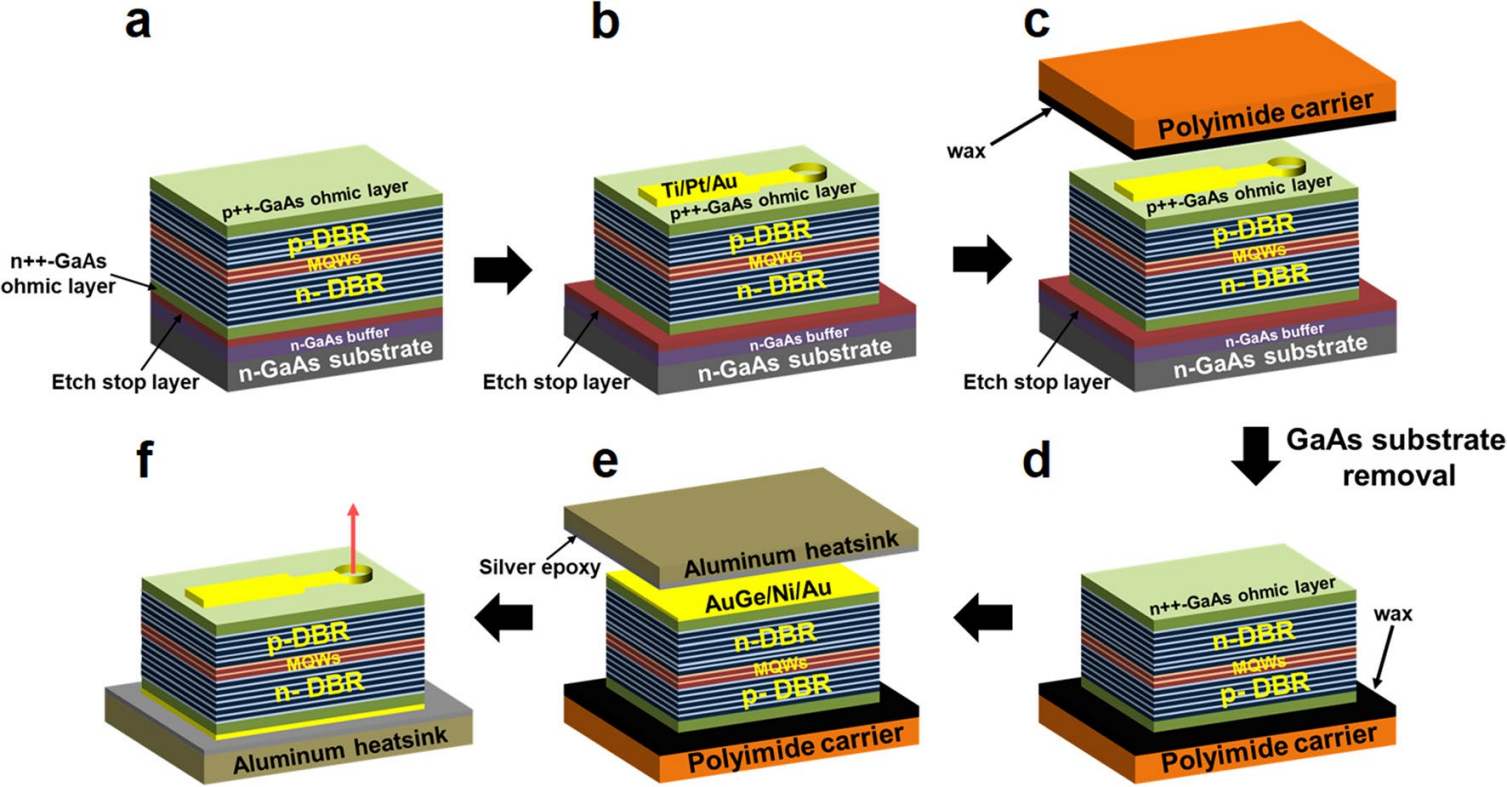
Double transfer technique to transfer the thin-film VCSELs onto aluminum heatsink. (**a**) P-on-n structures of thin-film 940-nm VCSELs are epitaxially grown in upright order via MOCVD. (**b**) The mesa etching is carried out by selectively removing the multiple layers of p- and n-DBR, and MQWs in order to isolate the VCSELs. Then, p-ohmic metal with a combination of Ti/Pt/Au is deposited on the highly p-doped GaAs layer. The 10-μm oxide-confined aperture size of each VCSEL was defined with selective oxidation of Al_x_Ga_1-x_As with high Al contents. (**c**) An adhesive wax is used for bonding the thin-film VCSELs to the polyimide carrier. (**d**) The etch stop layer is etched by acid solution after removing the 350-μm n-GaAs substrate in the NH_4_OH-based etchant. (**e**) The silver epoxy is uniformly applied on the aluminum heatsink, which is integrated with the wax-bonded thin-film VCSELs. (**f**) Top-emitting 940-nm thin-film VCSELs are successfully fabricated onto an aluminum heatsink after separating the thin-film VCSELs from the polyimide carrier.

Figure 3 shows the fabrication procedures of thin-film VCSELs onto an aluminum heatsink via a double-transfer technique. The double-transfer technique that transferred the thin-film VCSELs onto foreign substrates twice was carried out as follows: Fig. 3a shows that the p-on-n structures of top-emitting 940-nm thin-film VCSELs, including the etch stop layer and n-GaAs ohmic layer, are grown via MOCVD in upright order. Figure 3b shows the finished front processes of thin-film VCSELs, including mesa etching, p-metal deposition, and selective oxidation of Al_x_Ga_1-x_As before separating thin-film VCSELs from the growth substrate. A Mesa etching of n- and p-DBRs and MQWs was carried out in order to isolate the VCSELs. A grid pattern of p-ohmic contact with a combination of Ti/Pt/Au was deposited on the highly p-doped GaAs layer. The 10-μm oxide-confined aperture of each VCSEL was defined with selective oxidation of Al_x_Ga_1-x_As with high Al contents. Figure 3c shows the thin-film VCSELs mounted on a polyimide carrier by using adhesive wax. The adhesive wax was uniformly applied on the polyimide carrier at low temperature, which was bonded to the thin-film VCSELs with controlled pressure. The highly n-doped GaAs ohmic layer is exposed after removing the GaAs substrate and etch stop layer, as shown in Fig. 3d. The etch stop layer was selectively etched after the GaAs substrate removal, which prevented the NH_4_OH-based etchant from penetrating into the n-GaAs ohmic layer of the thin-film VCSELs during removal of the GaAs substrate. Figure 3e shows the wax-bonded thin-film VCSELs mounted onto an aluminum heatsink. After a combination of AuGe/Ni/Au was deposited on the n-GaAs ohmic layer, the wax-bonded thin-film VCSELs were integrated with silver-filled epoxy onto the aluminum heatsink. The wax-bonded thin-film VCSELs were completely bonded onto the aluminum heatsink during the curing of the silver-filled epoxy at 150 ℃ for one and a half hours. Figure 3f shows the top-emitting 940-nm thin-film VCSELs transferred onto the aluminum heatsink using the double-transfer technique. The polyimide carrier was separated by selectively removing the wax, thereby the top-emitting 940-nm thin-film VCSELs mounted onto the aluminum substrate were successfully fabricated.

### Operation of top-emitting 940-nm thin-film VCSELs with p-on-n polarity

**Figure 4:**
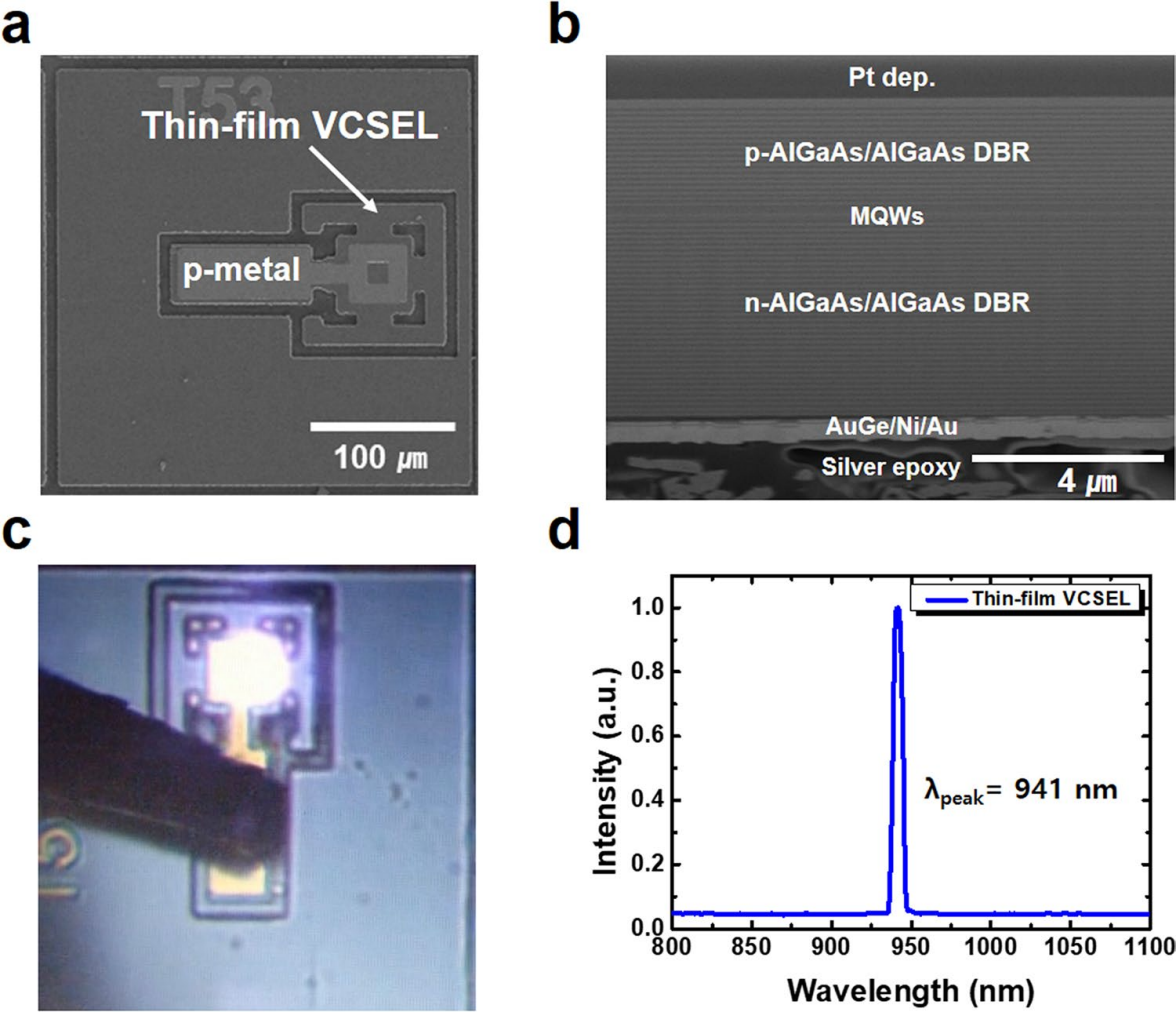
Fabrication of top-emitting 940-nm thin-film VCSELs. (**a**) Top-view and (**b**) cross-sectional SEM image of the fabricated thin-film VCSELs obtained from FIB milling **(c)** A microscopy image of the 940-nm top-emitting thin-film VCSELs (**d**) Spectroscopy of the emitting light from the 940-nm thin-film VCSELs.

Figure 4a shows a top-view scanning electron microscopy (SEM) image of the fabricated 940-nm top-emitting thin-film VCSELs onto the aluminum heatsink. The surface of the thin-film VCSELs mounted onto the aluminum heatsink was considerably smooth as shown in Supplementary Fig. S1. Figure 4b shows the cross-sectional SEM image, obtained using focused ion beam (FIB) milling, of the fabricated thin-film VCSELs where the n- and p-DBR layers as well as the MQWs are clearly visible. No significant dislocations of the transferred thin-film VCSELs onto the aluminum heatsink were observed in the VCSEL structure, demonstrating the feasible double-transfer technique for transferring the thin-film VCSELs onto foreign substrates. Figure 4c shows an optical microscopy image of the fabricated 940-nm top-emitting thin-film VCSELs onto the aluminum heatsink using a probe station. The full width at half maximum (FWHM) of lasing spectra at 8 mA was approximately 6.98 nm. The invisible laser emission of the 940-nm thin-film VCSELs at injection current of 8 mA was observed to be a purple beam using a conventional microscope^5^. As can be seen from Fig. 4d, the peak wavelength of the emitting light from the thin-film VCSELs was 941 nm which corresponded to the sharp reflectance dip near 940 nm shown in Supplementary Fig. S2.

**Figure 5:**
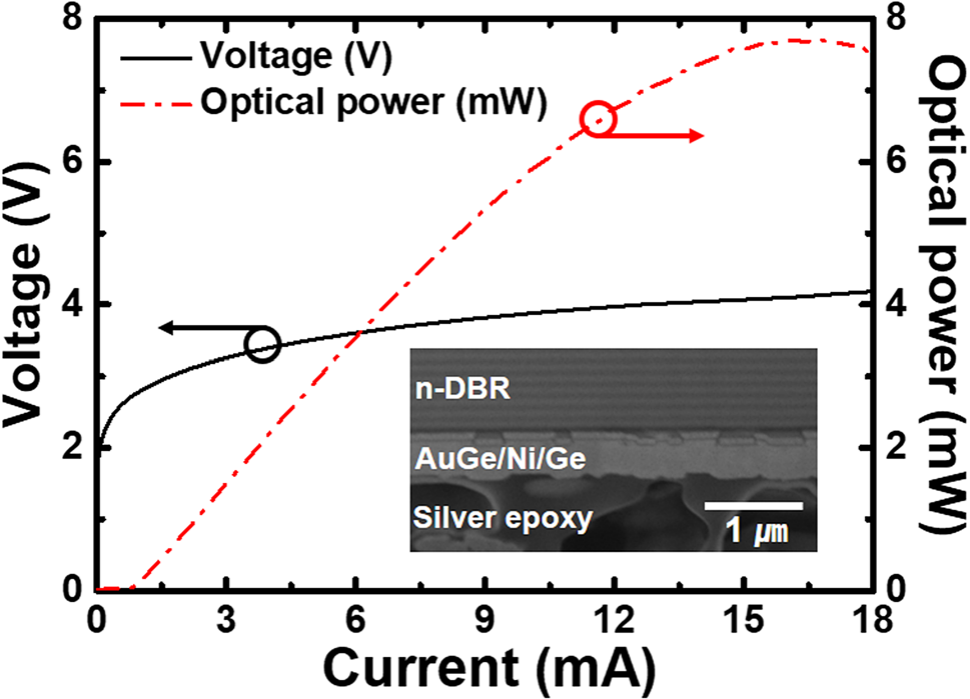
L–I–V characteristics of the top-emitting 940-nm thin-film VCSELs. The red lines (dash dot) are the optical power in the right y-axis, and the black lines are voltage in the left y-axis. The threshold current and voltage of the fabricated top-emitting 940 nm thin-film VCSELs are 1 mA and 2.8 V, respectively. The peak power of fabricated thin-film VCSELs is 7.7 mW at rollover current of 16.1 mA. The deformed area of n-ohmic contact layers in the SEM image could contribute to the increase in threshold voltage and steeper upward tilt to the slope of the I–V curve.

Figure 5 shows the L–I–V characteristics of the thin-film VCSELs onto the aluminum heatsink under continuous wave (CW) operation. The dot-dashed line indicates the threshold current of 1 mA. The peak power of the thin-film VCSELs was 7.7 mW at input current of 16.1 mA. A few deformations of contact layers, as shown in the SEM image of Fig. 5, might contribute to an increase in series resistance, resulting in a rather high threshold voltage of approximately 2.8 V and steeper upward tilt to the slope of the I-V curve of thin-film VCSELs.

## Discussion

The top-emitting 940-nm thin-film VCSELs transferred onto the aluminum heatsink were fabricated using the double-transfer technique, thereby the p-on-n polarity of the thin-film VCSELs remained unchanged. This approach does not require inverted growth which could have adverse effects on epi layers of the thin-film VCSELs. The care of the thin-film VCSELs was carefully taken during the double-transfer of the thin-film VCSELs onto foreign substrates. There was no discernible dislocation in the structures of the fabricated thin-film VCSELs, which successfully demonstrated the double-transfer technique for realization of high-power thin-film VCSELs mounted onto heatsinks. The adhesive wax was used to transfer the thin-film VCSELs onto the polyimide carrier with the removal of the GaAs substrate and etch stop layer. The etch stop layer was resistant to the NH_4_OH-based etchant that was used in order to etch the GaAs substrate, preventing the etchant from penetrating into epilayers of thin-film VCSELs. The wax-bonded thin-film VCSELs were integrated onto the aluminum heatsink using the silver-filled epoxy. The high thermal conductivity of silver-filled epoxy promoted efficient heat transfer to the aluminum heatsink, potentially contributing to extending the rollover point. The top-emitting 940-nm thin-film VCSELs were fabricated after separating the polyimide carrier from the wax-bonded thin-film VCSELs.

The threshold current and voltage of the top-emitting 940-nm thin-film VCSELs were 1 mA and 2.8 V, respectively. The series resistance of thin-film VCSEL was approximately 160 Ω, which could be degraded due to the irregular surface of the n-ohmic contact layer caused by the contraction stress of silver-filled epoxy during epoxy curing. This might contribute to the increase in the series resistance, resulting in the steeper curve of the I–V curve. A thermal resistance (*R*_*th*_) of the fabricated thin-film VCSELs can be extracted from the temperature rise with dissipated electrical input power as followed by an equation^8,11,22^.

1 $$R_{th} = \Delta{\text{T}}/\Delta{\text{P}}_{{{\text{diss}}}} = (\Delta\lambda /\Delta{\text{P}}_{{{\text{diss}}}} )/(\Delta\lambda /\Delta T)$$

where $\Delta$T is the temperature rise obtained from device active region, $\Delta$P_diss_ is the change of dissipated electrical power, and $\Delta$**λ** is the output wavelength shift.

**Figure 6:**
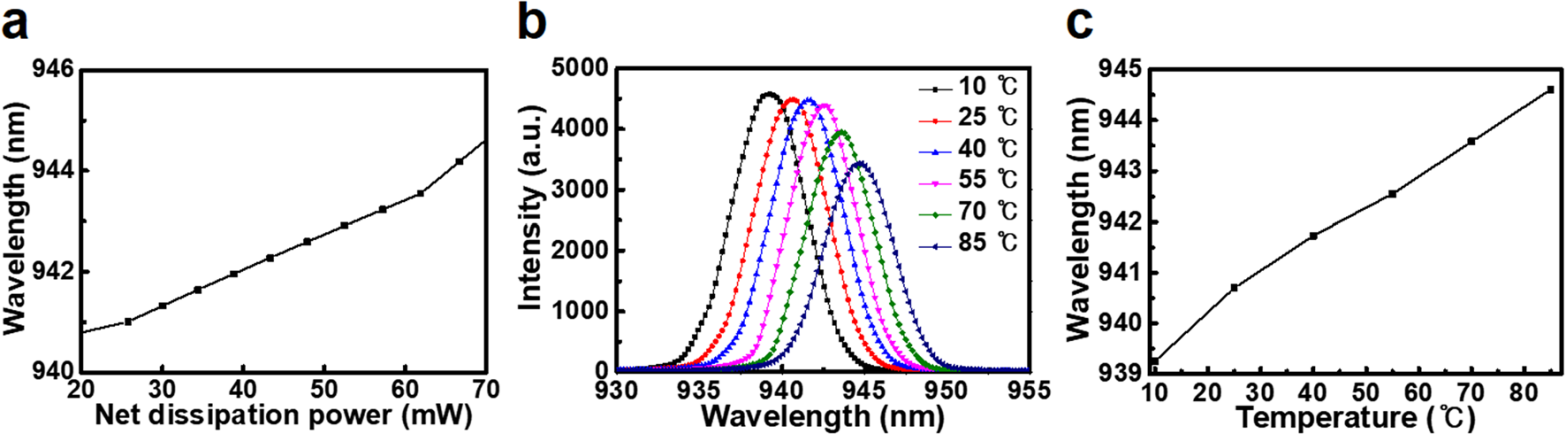
Thermal resistance (*R*_*th*_) of top-emitting 940-nm thin-film VCSEL. (**a**) Wavelength shift as a function of dissipated electrical input power. (**b**) Output spectrum of the thin-film VCSEL with the varied temperatures from 10 to 85 °C. (**c**) Thermal wavelength shift of the thin-film VCSEL for temperatures from 10 to 85 °C.

Figure 6a shows the wavelength shift as a function of dissipated electrical input power obtained from the fabricated thin-film VCSEL under continuous wave (CW) operation. The $\Delta$λ/$\Delta$P_diss_ value of the fabricated thin-film VCSEL was 0.0776 nm/mW.

Figure 6b shows the output spectrum of the fabricated thin-film VCSEL at input current of 8 mA under the controlled temperature from 10 to 85 °C. The output spectrum peak positon of the thin-film VCSEL showed the gradual red-shift from 939.3 to 944.6 nm with temperature rise. The output spectrum intensity of thin-film VCSEL was significantly degraded above 70 °C. The thermal wavelength shift as a function of the varied temperatures from 10 to 85 °C is shown in Fig. 6c. The $\Delta$·λ/$\Delta$·T value was 0.0713 nm/K. Therefore, the extracted *R*_*th*_ value of the fabricated thin-film VCSEL was 1088 K/W. The experimentally measured thermal resistance of the thin-film VCSELs mounted on the aluminum heatsinks was comparable to that of other thin-film VCSELs^8,23^.

Further performance enhancement of thin-film VCSELs could be realized in combination with direct transfer to ideal heatsinks by using metal electroplating instead of adhesive materials. The Au and Cu electroplating, in accordance with high thermal conductivities of 4.01 and 3.17 W/cm K^14^, respectively, can be used as efficient electrical and thermal passages, leading to further improved high-power thin-film VCSELs. Furthermore, this approach may reduce the thermal resistance induced by the irregular surface at bonding interface. Therefore, the fabricated top-emitting 940-nm thin-film VCSELs could exhibit superior thermal performance by using metal electroplating, leading to low-power consuming and high-power thin-film VCSELs.

In summary, the p-on-n structures of thin-film VCSELs were epitaxially grown via MOCVD in upright order. The double-transfer technique allowed top-emitting 940-nm thin-film VCSELs to keep the p-on-n polarity after transferring the thin-film VCSELs onto the aluminum heatsink. The threshold current and voltage of the fabricated thin-film VCSELs were estimated to be 1 mA and 2.8 V, respectively. The threshold voltage could be degraded due to the deformation of the n-GaAs ohmic layer. The peak power of the thin-film VCSELs was 7.7 mW at input current of 16.1 mA. We believe that the low-power consumption and high-power thin-film VCSELs fabricated using this double-transfer technique will enable the widespread use of thin-film VCSELs in three-dimensional depth sensing systems and modern lidar in automotive driving systems.

## Methods

### Epitaxial growth

The p-on-n structures of 940-nm thin-film VCSELs were grown via MOCVD in upright order as shown in Fig. 2. The used precursors were trimethylgallium (TMGa), trimethylaluminum (TMAl), trimethylindium (TMIn), arsine (AsH_3_), and phosphine (PH_3_). Silane (SiH_4_) was used as a dopant source for n-GaAs, whereas carbon (CBr_4_) was utilized for p-doping. The etch stop layer was inserted between the n-GaAs ohmic contact layer and n-GaAs buffer layer on n-GaAs (100) substrates. The three InGaAs/AlGaAs quantum wells were sandwiched between the 18-pair p-DBRs and 38-pair n-DBRs. The In and Al compostions of InGaAs/AlGaAs MQWs are 10.6 and 35%, respectively. The spectral reflectance spectrum exhibited a sharp reflectance dip near 940 nm caused by cavity resonance. (Supplementary Fig. S2). The Al_x_Ga_1-x_As with high Al fractions was included above the MQWs to define the 10-μm oxide-confined aperture size of thin-film VCSELs using selective oxidation of Al_x_Ga_1-x_As.

### Device characterization

The L–I–V characteristics of top-emitting 940-nm thin-film VCSELs with continuous wave (CW) operation were measured via a Keithley 2602B at room temperature. The Keithley current source was used to bias the thin-film VCSELs with electrical probing. A silicon photodiode (Hamamatsu, 2201 photodiode) was adopted in order to collect the 940-nm emission above the thin-film VCSELs. The emission spectrum of 940-nm thin-film VCSELs was investigated by using an Ocean optics Maya 2000 PRO Spectrometer.

## Supplementary Information

Supplementary Information.
