# Supplementary material for: Top-emitting 940-nm thin-film VCSELs transferred onto aluminum heatsinks
Source: Sci Rep. 2022 Jan 12;12:565. doi: 10.1038/s41598-021-04625-6 (PMC8755793; doi:10.1038/s41598-021-04625-6)
Supplement: Supplementary file 1 — Supplementary Information. [file 41598_2021_4625_MOESM1_ESM.docx]

**Supplementary Information:**

**Top-emitting 940-nm thin-film VCSELs
transferred onto aluminum heatsinks**

**Sunghyun Moon^1^, Yeojun Yun^1^, Minhyung Lee^1^, Donghwan Kim^3^**, **Wonjin Choi^3^**, **Ji-Yong** **Park^2^ and Jaejin Lee^1,*^**

^1^Department of Electrical and Computer Engineering, Ajou University, Suwon, 16499, South Korea

^2^ Department of Physics and Department of Energy Systems Research, Ajou University, Suwon, 16499, South Korea

^3^RayIR Corporation, LTD, 156 Gwanggyo-ro, Yeongtong-gu, Suwon, 16506, South Korea

^*^Corresponding author: [jaejin@ajou.ac.kr](mailto:jaejin@ajou.ac.kr)

**Surface roughness of the fabricated top-emitting thin-film VCSELs**


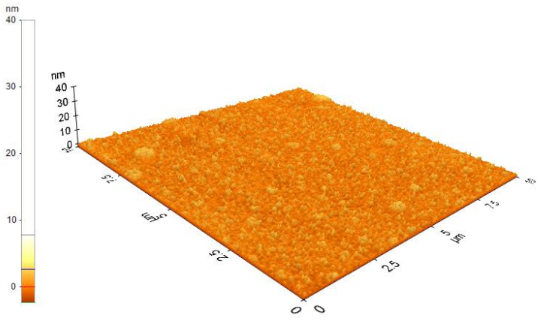
Supplementary Figure S1 shows 10 × 10-μm^2^ atomic force microscopy (AFM) image of the fabricated thin-film VCSEL onto an aluminum heatsink. The fabricated thin-film VCSEL exhibited smooth surface with a root-mean-square (*RMS*) value of 0.774 nm.

**Supplementary Figure S1. The 10 × 10-μm^2^ AFM image of the fabricated thin-film VCSEL onto an aluminum substrate.** The fabricated thin-film VCSEL showed smooth surface with a *RMS* value of 0.774 nm.

**Reflectance spectrum of 940-nm thin-film VCSELs**

**
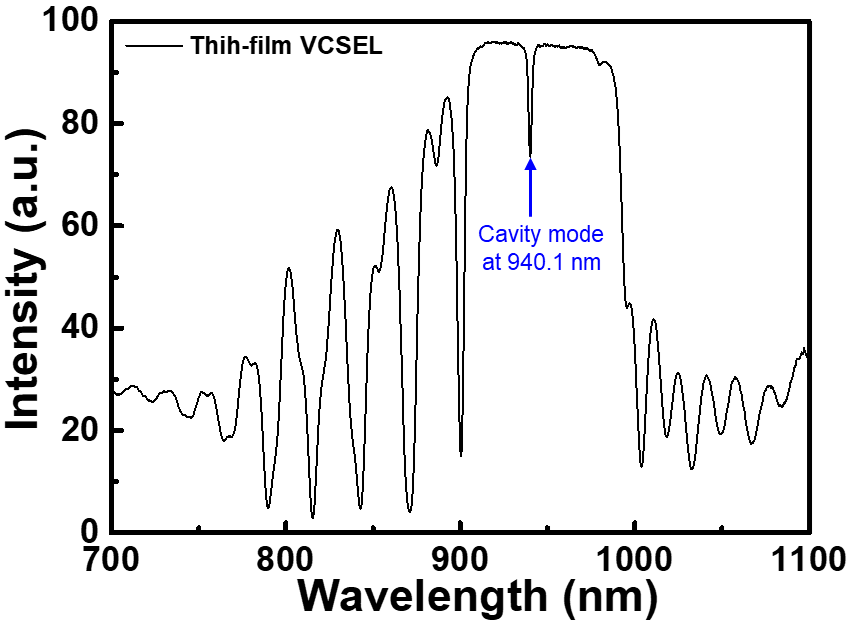
**The reflectance spectrum of 940-nm thin-film VCSELs with p-on-n structures, including 38-pair n-DBRs, 18-pair p-type DBRs, and 3-pair MQWs, is shown in Fig. S2. The bottom n-DBRs with 38 pairs exhibited maximal reflectivity, whereas the top p-DBRs with lower numbers than n-DBRs were designed to ensure the light emission of the thin-film VCSELs. The cavity mode (CM) dip in the reflectance spectrum of thin-film VCSELs was centered at 940.1 nm.

**Supplementary Figure S2. Reflectance spectrum of 940-nm thin-film VCSELs with p-on-n structures.** The epitaxial structures of thin-film VCSELs consist of top p-DBR and bottom n-DBR mirrors as well as an active gain region with InGaAs−AlGaAs MQWs.
